# Expert Search Strategies: The Information Retrieval Practices of Healthcare Information Professionals

**DOI:** 10.2196/medinform.7680

**Published:** 2017-10-02

**Authors:** Tony Russell-Rose, Jon Chamberlain

**Affiliations:** ^1^ UXLabs Ltd Guildford United Kingdom; ^2^ School of Computer Science and Electronic Engineering University of Essex Colchester United Kingdom

**Keywords:** review, surveys and questionnaires, search engine, information management, information systems

## Abstract

**Background:**

Healthcare information professionals play a key role in closing the knowledge gap between medical research and clinical practice. Their work involves meticulous searching of literature databases using complex search strategies that can consist of hundreds of keywords, operators, and ontology terms. This process is prone to error and can lead to inefficiency and bias if performed incorrectly.

**Objective:**

The aim of this study was to investigate the search behavior of healthcare information professionals, uncovering their needs, goals, and requirements for information retrieval systems.

**Methods:**

A survey was distributed to healthcare information professionals via professional association email discussion lists. It investigated the search tasks they undertake, their techniques for search strategy formulation, their approaches to evaluating search results, and their preferred functionality for searching library-style databases. The popular literature search system PubMed was then evaluated to determine the extent to which their needs were met.

**Results:**

The 107 respondents indicated that their information retrieval process relied on the use of complex, repeatable, and transparent search strategies. On average it took 60 minutes to formulate a search strategy, with a search task taking 4 hours and consisting of 15 strategy lines. Respondents reviewed a median of 175 results per search task, far more than they would ideally like (100). The most desired features of a search system were merging search queries and combining search results.

**Conclusions:**

Healthcare information professionals routinely address some of the most challenging information retrieval problems of any profession. However, their needs are not fully supported by current literature search systems and there is demand for improved functionality, in particular regarding the development and management of search strategies.

## Introduction

### Background

Medical knowledge is growing so rapidly that it is difficult for healthcare professionals to keep up. As the volume of published studies increases each year [1], the gap between research knowledge and professional practice grows [2]. Frontline healthcare providers (such as general practitioners [GPs]) responding to the immediate needs of patients may employ a Web-style search for diagnostic purposes, with Google being reported to be a useful diagnostic tool [3]; however, the credibility of results depends on the domain [4]. Medical staff may also perform more in-depth searches, such as rapid evidence reviews, where a concise summary of what is known about a topic or intervention is required [5].

Healthcare information professionals play the primary role in closing the gap between published research and medical practice, by synthesizing the complex, incomplete, and at times conflicting findings of biomedical research into a form that can readily inform healthcare decision making [6]. The systematic literature review process relies on the painstaking and meticulous searching of multiple databases using complex Boolean search strategies that often consist of hundreds of keywords, operators, and ontology terms [7] (Textbox 1).

**An example of a multi-line search strategy.**

1. Attention Deficit Disorder with Hyperactivity/
2. adhd
3. addh
4. adhs
5. hyperactiv$
6. hyperkin$
7. attention deficit$
8. brain dysfunction
9. or/1-8
10. Child/
11. Adolescent/
12. child$ or boy$ or girl$ or schoolchild$ or adolescen$ or teen$ or “young person$” or “young people$” or youth$
13. or/10-12
14. acupuncture therapy/or acupuncture, ear/or electroacupuncture/
15. accupunct$
16. or/14-15
17. 9 and 13 and 16

Performing a systematic review is a resource-intensive and time consuming undertaking, sometimes taking years to complete [8]. It involves a lengthy content production process whose output relies heavily on the quality of the initial search strategy, particularly in ensuring that the scope is sufficiently exhaustive and that the review is not biased by easily accessible studies [9].

Numerous studies have been performed to investigate the healthcare information retrieval process and to better understand the challenges involved in strategy development, as it has been noted that online health resources are not created by healthcare professionals [10]. For example, Grant [11] used a combination of a semi-structured questionnaire and interviews to study researchers’ experiences of searching the literature, with particular reference to the use of optimal search strategies. Sampson et al [12] used a combination of a Web-based survey and peer review forums to investigate what elements of the search process have the most impact on the overall quality of the resulting evidence base. Similarly, Gillies et al [13] used an online survey to investigate the review, with a view to identifying problems and barriers for authors of Cochrane reviews. Ciapponi and Glujovsky [14] also used an online survey to study the early stages of systematic review.

No single database can cover all the medical literature required for a systematic review, although some are considered to be a core element of any healthcare search strategy, such as MEDLINE [15], Embase [16], and the Cochrane Library [17]. Consequently, healthcare information professionals may consult these sources along with a number of other, more specialized databases to fit the precise scope area [18].

A survey [1] of online tools for searching literature databases using PubMed [19], the online literature search service primarily for MEDLINE, showed that most tools were developed for managing search results (such as ranking, clustering into topics and enriching with semantics). Very few tools improved on the standard PubMed search interface or offered advanced Boolean string editing methods in order to support complex literature searching.

### Objective

To improve the accuracy and efficiency of the literature search process, it is essential that information retrieval applications (in this case, databases of medical literature and the interfaces through which they are accessed) are designed to support the tasks, needs, and expectations of their users. To do so they should consider the layers of context that influence the search task [20] and how this affects the various phases in the search process [21]. This study was designed to fill gaps in this knowledge by investigating the information retrieval practices of healthcare information professionals and contrasting their requirements to the level of support offered by a widely used literature search tool (PubMed).

The specific research questions addressed by this study were (1) How long do search tasks take when performed by healthcare information professionals? (2) How do they formulate search strategies and what kind of search functionality do they use? (3) How are search results evaluated? (4) What functionality do they value in a literature search system? (5) To what extent are their requirements and aspirations met by the PubMed literature search system?

In answering these research questions we hope to provide direct comparisons within other professions (eg, in terms of the structure, complexity, and duration of their search tasks).

## Methods

### Online Survey

The survey instrument consisted of an online questionnaire of 58 questions divided into 5 sections. It was designed to align with the structure and content of Joho et al’s [22] survey of patent searchers and wherever possible also with Geschwandtner et al’s [23] survey of medical professionals to facilitate comparisons with other professions. The following were the 5 sections: (1) Demographics, the background and professional experience of the respondents; (2) Search tasks, the tasks that respondents perform when searching literature databases; (3) Query formulation, the techniques respondents used to formulate search strategies; (4) Evaluating search results, how respondents evaluate the results of their search tasks; and (5) Ideal functionality for searching databases, any other features that respondents value when searching literature databases.

The survey was designed to be completed in approximately 15 minutes and was pre-tested for face validity by 2 health sciences librarians.

Survey respondents were recruited by sending an email invitation with a link to the survey to 5 healthcare professional association mailing lists that deal with systematic reviews and medical librarianship: Lis-Medical [24], clinical librarians [25], evidence-based health [26], expert searching [27], and the Cochrane Information Retrieval Methods Group (IRMG) [28]. It was also sent directly to the members of the Chartered Institute of Library and Information Professionals (CILIP) Healthcare Libraries special interest group [29]. The recruitment message and start page of the survey described the eligibility criteria for survey participants, expected time to complete the survey, its purpose, and funding source.

The survey (Multimedia Appendix 1) was conducted using SurveyMonkey, a Web-based software application [30]. Data were collected from July to September 2015. A total of 218 responses were received, of which 107 (49.1%, 107/218) were complete (meaning all pages of the survey had been viewed and all compulsory questions responded to). Only complete surveys were examined. Since the number of unique individuals reached by the mailing list announcements is unknown, the participation rate cannot be determined.

Responses to numeric questions were not constrained to integers as a pilot survey had shown that respondents preferred to put in approximate and/or expressive values. Text responses corresponding to numerical questions (questions 14 to 22 and 32 to 38; 16 in total) were normalized as follows: (1) when the respondent specified a range (eg, 10 to 20 hours), the midpoint was entered (eg, 15 hours); (2) when the respondent indicated a minimum (eg, 10 years and greater), the minimum was entered (eg, 10 years); and (3) when the respondent entered an approximate number (eg, about 20), that number was entered (eg, 20).

After normalizing, 8.29% (142/1712) responses contained no numerical data and 21.61% (370/1712) responses were normalized.

### Evaluation of PubMed

An evaluation of the PubMed search system was performed using online documentation [31], best practice advice [32], and direct testing of the interface using Boolean commands. In addition to the search portal, users can register to My NCBI which provides additional functionality for saving search queries, managing results sets, and customizing filters so this was included in the comparison. The mobile version of PubMed, PubMed Mobile [33], does not offer extended functionality so it was not considered in the evaluation. Although beyond the scope of this study, information seeking by healthcare practitioners on hand-held devices has been shown to save time and improve the early learning of new developments [34].

## Results

### Demographics

**Figure 1:**
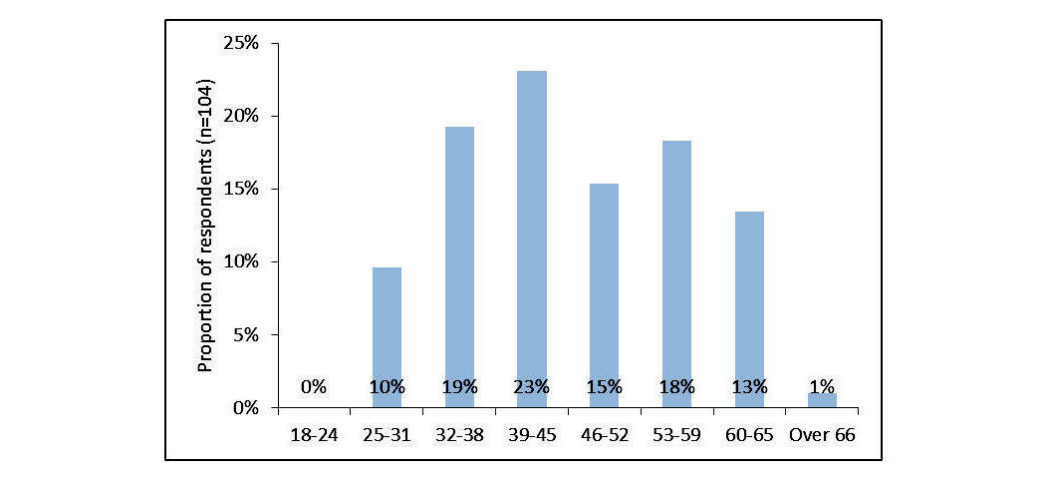
Age of respondents.

Of the respondents, 89.3% (92/103) were female. Their ages were distributed bi-modally, with peaks at 39 to 45 and 53 to 59, with a conflated average age of 46.0 (SD 10.9, N=104) (Figure 1).

The mean time for respondents' experience in their profession was 16.6 years (SD 10.0), greater than their 12.0 (SD 9.0) years of experience in the review of scientific literature (N=107, *P*<.01, paired *t* test). Most respondents worked full time (78.5%, 84/107) and the commissioning agents for their searches were predominantly internal (ie, within the same organization [72.9%, 78/107]).

The majority of respondents were either based in the UK (51.4%, 55/107), in the US (27.1%, 29/107), or in Canada (7.5%, 8/107). The remaining respondents were from Australia (2.8%, 3/107), Netherlands, Norway, and Germany (1.9% each, 2/107), and Denmark, Singapore, Uruguay, South Africa, Belgium, and Ireland (0.9% each, 1/107). All (100.0%, 107/107) respondents stated that the language they used most frequently for searching was English; however, 6.5% (7/107) stated that they did not use English most frequently for communication in their workplace.

The majority of respondents (81.3%, 87/107) worked in organizations that provide systematic reviews. These organizations also provided other services including reference management (72.0%, 77/107), rapid evidence reviews (63.6%, 68/107), background reviews (60.7%, 65/107), and critical appraisals (52.3%, 56/107).

**Table 1:** Effort to complete search tasks and evaluate results.

Task	Minimum (IQR^a^)	Average (IQR)	Maximum (IQR)
Search time per document collection/database, minutes	20 (10-30)	60 (27.5-150)	228 (86-480)
Search task completion time, hours	1 (0.5-2)	4 (2-6.5)	14 (7-30)
Strategy lines per search task, n	5 (2.8-10)	15 (9.1-30)	59 (30-105)
Results examined from a search task, n	10 (5-32)	175 (75-500)	850 (400-5250)
Time to assess relevance of a single result/document, minutes	1 (0.5-2)	3 (1-5)	10 (5-25)
Ideal number of search results per search task, n	0	100	10,000

^a^IQR: interquartile range.

### Search Tasks

We considered a search task in this context to be the creation of one or more strategy lines to search a specific collection of documents or database, with task completion resulting in a set of search results that will be subject to further analysis. The output of this process is the search strategy, which is often published as part of the search documentation. This rationalization is in line with a healthcare information professionals’ understanding but the complexity of search tasks in this domain is discussed in more detail later.

The time spent formulating search strategies, the amount of time respondents spend completing search tasks, and the number of strategy lines they use is shown in Table 1. Respondents were asked to estimate a minimum, average, and maximum for each of these measures, and the values reported here are the medians of each with the interquartile range (IQR) shown in brackets (in the form Q1 to Q3). The final row shows the minimum, average, and maximum answers to the question: “What would you consider to be the ideal number of results returned for a typical search task?” On average, it takes 60 minutes to formulate a search strategy for a document collection, with the search task taking 4 hours to complete, and the final strategy consisting of 15 lines.

**Figure 2:**
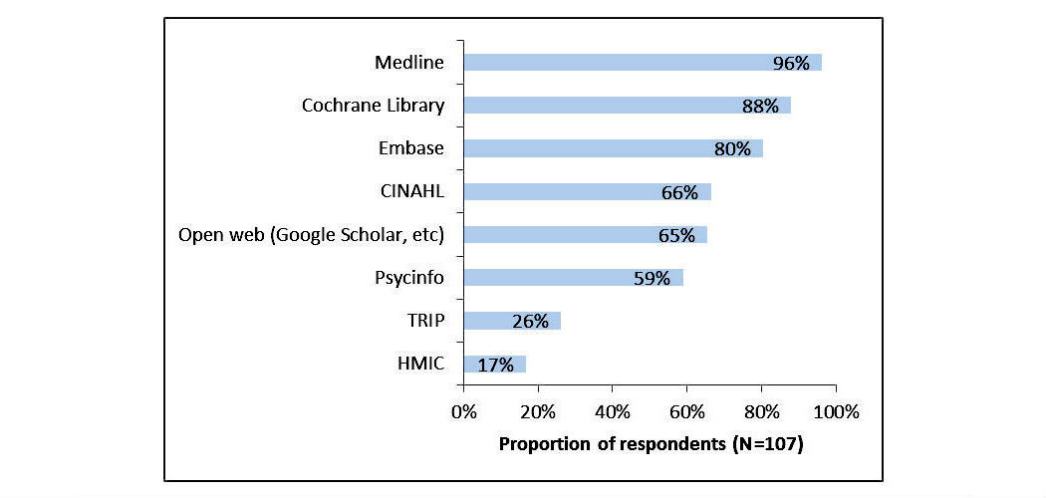
Data sources most frequently searched.

The data sources most frequently searched were MEDLINE (96.3%, 103/107), the Cochrane Library (87.9%, 94/107), and Embase (80.4%, 86/107) (Figure 2).

The majority of respondents (86.9%, 93/107) used previous search strategies or templates at least sometimes, suggesting that the value embodied in them is recognized and should be re-used wherever possible. In addition, most respondents (89.7%, 96/107) routinely share their search strategies in some form, either with colleagues in their workgroup, more broadly within their organization, or in some other capacity (eg, with clients or as part of a published review).

### Query Formulation

**Figure 3:**
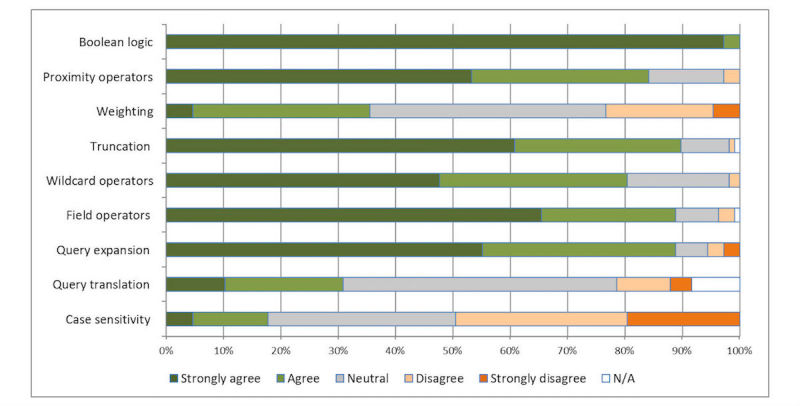
Importance of query formulation functionality.

We examined the mechanics of the query formulation process by asking respondents to indicate a level of agreement to statements using a 5-point Likert scale ranging from 1 (strong disagreement) to 5 (strong agreement). The results are shown in Figure 3.

When asked which taxonomies are regularly used, 74.8% (80/107) of respondents indicated they used MeSH, 45.8% (49/107) Emtree, and 18.9% (20/107) CINAHL headings.

When asked which combination of techniques they used to create their search strategies, 44.9% (48/107) stated they used a form-based query builder, 41.1% (44/107) did so manually on paper, and 40.2% (43/107) used a text editor. Only 9.3% (10/107) used some form of visual query builder.

### Evaluating Search Results

Respondents indicated that the ideal number of results returned for a search task would be 100 documents, yet in practice they evaluate more than this (a median of 175 documents; Table 1). The ideal number of results and the actual number of results evaluated are strongly correlated (N=66, ρ=.661 [Spearman rank correlation]). The average time to assess relevance of a single document was 3 minutes.

**Figure 4:**
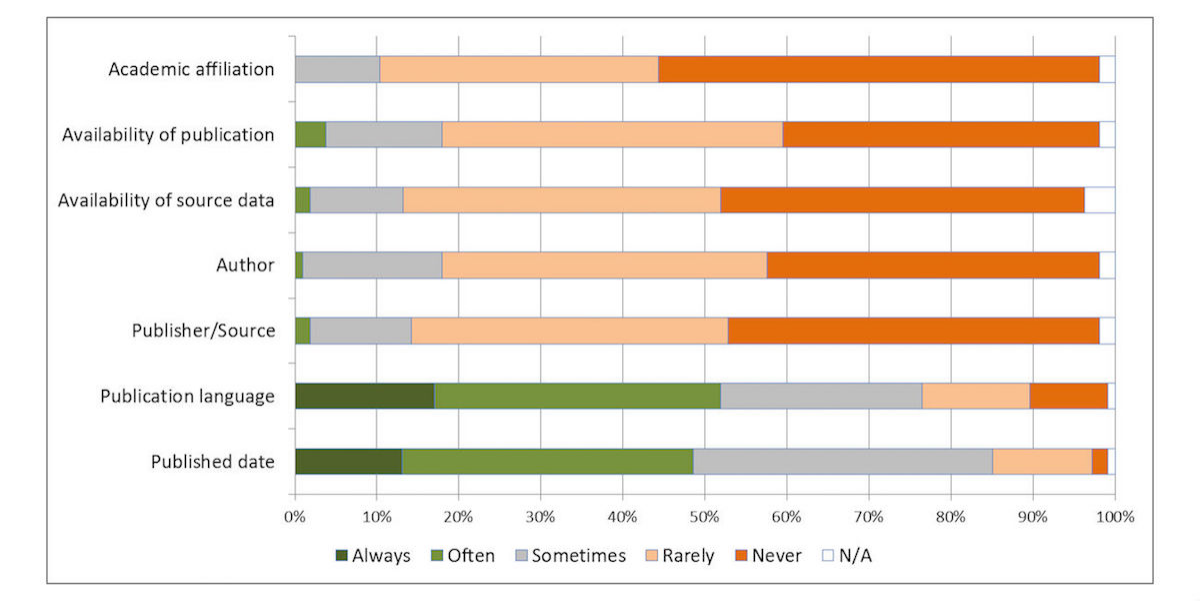
Usage of restriction criteria.

Respondents were asked to indicate on a 5-point Likert scale how frequently they use search limits and restriction criteria to narrow down results. The results are shown in Figure 4.

We also examined respondents’ strategies for examining the search results. The most popular approaches were to “start with the result that looked most relevant” (54.2%, 58/107) or simply “select the first result” (23.4%, 25/107). No respondent suggested selecting the “most trustworthy source.”

**Figure 5:**
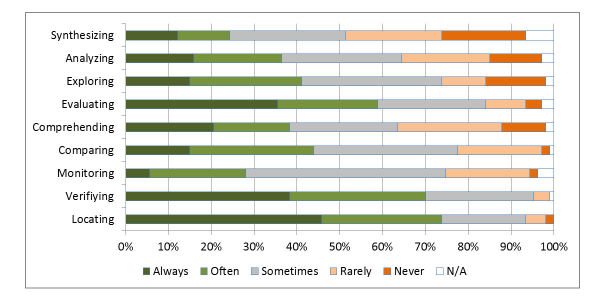
Activities that respondents engage in when completing a search task.

Respondents were asked what types of activities [35] they typically engaged in whilst completing their search task (Figure 5). “Locating, verifying, and evaluating results” were the most common activities (see Multimedia Appendix 1 for the full description of each activity, as provided to the respondents).

**Figure 6:**
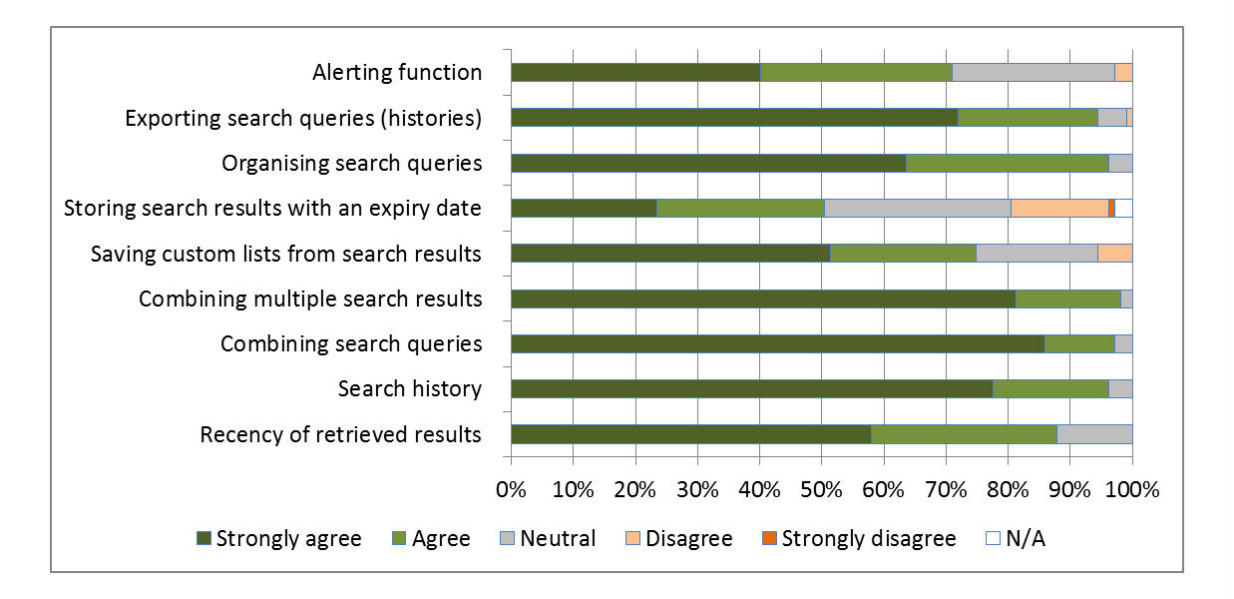
Ideal features of a literature search system.

### Ideal Functionality for Searching Databases

We also examined other features related to search management, organization, and history that respondents value when performing search tasks. Respondents were asked to indicate a level of agreement to a statement using a 5-point Likert scale ranging from 1 (strong disagreement) to 5 (strong agreement). The results are shown in Figure 6.

## Discussion

Here, the implications of the results with verbatim responses to the question “How could the process of creating and managing search strategies be improved for you?” are discussed and the findings are contextualized in relation to the PubMed literature search system.

### Search Tasks

The respondents showed they invest considerable amounts of time performing search tasks and writing search strategies. The time to search a document collection (60 minutes) indicated that their search strategies were more complex to create than most literature search queries, given that 90% of individual queries on PubMed take less than 5 minutes [36]. It is also longer than diagnostic Web searches typical of front-line healthcare professionals (only 14% of medical practitioners reported spending more than 40 minutes on this search task) [23].

This search effort is often recycled and routinely shared indicating a need for facilities to manage and share strategies such as: “...being able to download, share, remix, transfer and translate search strategies.” PubMed does not offer the ability to share search queries, only the results in the form of citation Collections.

### Query Formulation

The results in Figure 3 suggest 2 observations regarding how healthcare information professionals formulate queries. Firstly, the scores suggest a willingness to adopt a wide range of search functionality to complete search tasks. This represents a marked contrast to the behavior of typical Web searchers who rarely, if ever, use any advanced search functionality [37]. Secondly, the use of Boolean logic was shown to be the most important feature, closely followed by the use of synonyms and related terms. A number of other syntactic features, notably proximity operators, truncation, and wildcarding, all scored highly, reflecting the need for fine control over search strategies. Field operators were also judged to be important, reflecting the structured nature of the document collections that are searched. Query expansion (ie, terms are expanded to include synonyms) scored highly, underlining the key role that controlled vocabularies such as MeSH play in forming effective search strategies (75% of respondents were familiar with using MeSH headings) and a requirement for, ideally, with “one universal thesaurus of medical terminology for all databases”.

PubMed offers most of the query formulation functionality described in Figure 3, either through explicit Boolean queries or through related functionality. Simple keyword queries are converted into Boolean queries by using the AND operator, attempting to automatically align the keywords with MeSH terms (called Automatic Term Mapping) and expanding the query to match all search phrases. Boolean operators OR and NOT are also accepted. Users can search specific fields by using square brackets after the search term (such as for searching within abstract, author, title, etc). Spelling correction and phrase completion are offered as the user types into the textbox. Wildcard and truncation is partially supported by allowing right-truncation only (ie, child*) would return results for children and childhood. Proximity operators are not supported; however, PubMed offers a list of related articles derived from a word-weighted algorithm [38]. Search queries can also be made in multiple languages (although the only non-English data in PubMed is currently limited to the “transliterated title” field). The only functionality PubMed does not appear to offer is weighting search terms and case sensitivity, both of which were rated as the least important functionality by respondents of the survey. This highlights the difference between comprehensive searches for literature as required for a review compared to more general Web searches where relevance ranking with semi-automatic methods would be considered more important.

A previous study has shown that as many as 90% of published strategies contained an error [39] and that reporting of strategies is commonly not in line with best practice [40]. A number of respondents suggested that healthcare information professionals need advanced query formulation support to help them with search tasks (Textbox 2).

**Examples of search functionality that require advanced query formulation support.**

Search functionality

- Syntax checking: “…automate checking of parentheses, operators and field codes…”
- Truncation: “Wildcards at beginning of words; wildcard within a word (to replace a single or multiple letters eg, $sthetic or wom$n”
- Misspellings: “…account for misspellings…” and “UK/American spelling…”
- Proximity: “…interpreting proximity within sentence rather than crossing punctuation limits.”
- Term frequency and location: “…terms in the first and/or last sentence of the abstract only”
- Negation: “…a negation that doesn't exclude articles where the negated concept is preceded by a negation. ex: NOT “palliative care” will exclude abstracts with sentences like this 'in this study we didn't take in account palliative care'”

PubMed allows users to build queries in stages using an HTML form to capture the query, then listing previous queries below in order for the user to make composite strategies of increasing complexity. Given that the average number of strategy lines required for a search task here was 15, this method of query construction can get increasingly complex and difficult for the user to understand and manipulate. Only 5.7% (10/176) of survey respondents reported using a visual query builder, an indication that there is very little support for healthcare information professionals in the intuitive construction of complex search queries. They also indicated a desire for advanced editing functionality, in particular:

move search lines up and down the history…

being able to add tags or descriptions to search strategies, ability to sort by name, topic or date...

take notes about why you added terms, syntax, etc.

It is clear that respondents commonly work across multiple platforms, in particular MEDLINE, Cochrane, and Embase, and this is in line with findings from previous research [41]. There is therefore a need for standardization and consistency between suppliers: “A service that could map search strategy between databases would save a lot of time.”

### Evaluating Search Results

The figure of 100 average (median) ideal search results masks the non-parametric nature of the data; the number of search results obtained may vary considerably depending on the topic and body of literature available in that domain [41]. Healthcare information professionals may adjust their expectations of sensitivity (or recall) in relation to their searches, depending on the need for coverage and inclusiveness. Clearly the ideal response from a search is more nuanced than a single figure can convey; however, respondents indicated that they find more results from their searches than they would ideally like to evaluate. This may be the result of an abundance of published research or that the search parameters are not restrictive enough to return an appropriate number of results.

The time to assess each result (3 minutes) seems short when considering the length of some of the documents that will be analyzed. However, the search task is the first stage of a much longer process in which the retrieved documents are exposed to further phases of evaluation (Figure 5). In this context, the time to assess relevance may reflect the dynamics of the initial sift, which is a much smaller fraction of the overall attention given to a document.

Publication date was considered to be the most important results filtering criteria, followed by publication language (Figure 4); however, other criteria were not considered important in the restriction of results. Certain respondents mentioned other criteria they use including publication type, study scope (eg, human only), study design, age range, and gender. All filtering and restriction criteria mentioned here can be used to narrow down results in PubMed.

The fact that no respondent valued sorting by most trustworthy source contrasts sharply with the strategies used in another study of the healthcare profession [23]. This most likely reflects the difference between the largely curated (and to some extent implicitly trusted) databases referred to in our study (MEDLINE, Embase, and the Cochrane Library) and the relatively uncontrolled Web resources used in Geschwandtner’s study.

### Ideal Functionality

Respondents scored all options of ideal functionality highly, indicating a general desire for advanced search functionality. Combining search queries and combining search results were rated as the most important, reflecting the current paradigm for building search queries (ie, the line-by-line strategy building approach offered by most databases including PubMed). The participants rated the ability to export search queries (histories) highly, reflecting their need to publish completed search strategies as part of their professional practice.

All of the functionality that is described in Figure 6 as being desired by healthcare information professionals is available through PubMed, either directly or by registering as a free user of My NCBI. It is therefore surprising that the verbatim responses from the respondents indicated that typical systems fall short in terms of their needs.

One reason may be that PubMed attempts to cater for a wide range of user knowledge (approximately one third of PubMed users are not domain experts [42]) and search expertise, from simple keyword queries to complex search strategies. Query log analysis has shown a difference between how users of different skills perform on PubMed [43] and PubMed attempts to accommodate all their needs in one interface. One example of this compromise is the lack of truncation and proximity operators, which may be exactly what is required by a healthcare information professional performing a systematic review for a topic with few articles.

### Limitations

A limitation of this survey is the sample size compared to some surveys of healthcare information professionals [13,14]; however, engagement with professionals in this sector has been shown to be challenging, with lower participation rates reported elsewhere [11,12]. We believe the completion rate of the survey (49.1%, 107/218) is high for a survey of this length (approximately 15 minutes); however, greater participation could have been achieved with a shorter, more targeted survey. We acknowledge that the lack of control over distribution and that it was administered in English only may introduce selection bias. The demographics of this survey have a similar distribution to a larger survey of healthcare information professionals [44] (95% females compared to 86% reported here, with an average age of 47.2 compared to 46.0 here), an indication that the sampled population may be representative of the profession.

A further limitation of this study is whether respondents fully understood our distinction between search tasks and search strategies (which follows the precedent of previous survey designs and hence facilitates direct comparison with their results). An additional evaluation of other literature search tools (such as Ovid) would have provided a more extensive survey of functionality available to healthcare information professionals; however, as PubMed was the most frequently used by the respondents it is more representative of the tools they have at their disposal. A full survey of free and subscription search tools available in healthcare would be useful future work. Despite these limitations we believe the research provides valuable insight into the requirements of healthcare information professionals.

### Conclusions

This paper summarizes the results of a survey of the information retrieval practices of healthcare information professionals, focusing in particular on the process of search strategy development. Our findings suggest that they routinely address some of the most challenging information retrieval problems of any profession, but current literature search systems offer only limited support for their requirements. The functionality offered by PubMed goes some way toward meeting those needs, but is compromised by the need to serve all types of users who may not require the same degree of fine control over their search strategies. In particular, there is a need for improved functionality regarding the management of search strategies and the ability to search across multiple databases.

The results of this study will be used to inform the development of future retrieval systems for healthcare information professionals and for others performing healthcare-related search tasks.

## Appendix

Multimedia Appendix 1 Survey Instrument.
