# Supplementary material for: Expert Search Strategies: The Information Retrieval Practices of Healthcare Information Professionals
Source: JMIR Med Inform. 2017 Oct 2;5(4):e33. doi: 10.2196/medinform.7680 (PMC5643841; doi:10.2196/medinform.7680)
Supplement: Multimedia Appendix 1 [file medinform_v5i4e33_app1.pdf]

The Information-Seeking Behaviour of Healthcare Information Professionals

Multimedia Appendix 1: Survey Instrument

UXLabs is participating in an InnovateUK-funded project investigating the use of complex search strategies in the workplace, with the aim of producing requirements for the design of next generation search tools. By collaborating in this research you can help make the process of creating and managing complex search queries easier and more efficient.

Complex search strategies can include Boolean queries such as this:

("etiology"[Subheading] OR "etiology"[All Fields] OR "causes"[All Fields] OR "causality"[MeSH Terms] OR "causality"[All Fields]) AND ("somnambulism"[MeSH Terms] OR "somnambulism"[All Fields] OR ("sleep"[All Fields] AND "walking"[All Fields]) OR "sleep walking"[All Fields])

Or incremental, line-by-line strategies like this:

1 Attention Deficit Disorder with Hyperactivity/
2 adhd
3 addh
4 adhs
5 hyperactiv$
6 hyperkin$
7 attention deficit$
8 brain dysfunction
9 or/1-8
10 Child/
11 Adolescent/
12 child$ or boy$ or girl$ or schoolchild$ or adolescen$ or teen$ or "young person$" or "young people$" or youth$
13 or/10-12
14 acupuncture therapy/or acupuncture, ear/or electroacupuncture/
15 accupunct$
16 or/14-15
17 9 and 13 and 16

This survey is 7 pages long and should take approximately 15 minutes to complete.

* indicates a question that must be completed

How do professionals search? Share your experience of search strategy formulation.

* 1. Does your work involve the review of scientific/professional literature?

Yes
No

Your background and experience in literature review.

* 2. Are you a full-time or part-time employee?
Full-time
Part-time

* 3. Who are your main clients?
Internal (from the same organisation)
External (from a different organisation)
Both

* 4. Where is your organisation (local office) located?

5. What is your role within your organisation?

* 6. How many years have you worked in the healthcare / information industry?

* 7. For how many years has your work involved the review of scientific literature?

8. What language do you use for **searching** MOST FREQUENTLY?

9. What language do you use for **communication** MOST FREQUENTLY?

Search Tasks

10. What services does your organization provide? (select all that apply)
Systematic review
Rapid evidence review
Background review
Guideline production
Future trends analysis/Horizon scanning
Reference management
Critical appraisal

* 11. How often do you perform these search tasks as part of your daily practice?
Always Often Sometimes Rarely Never N/A
Proactive monitoring of the state-of-the-art
Adhoc specific requests
Searching for specific incidents
Knowledge awareness
Other (please specify)

12. What other search tasks do you perform regularly as part of your daily practice?

13. For the search task you perform MOST FREQUENTLY, what data sources do you use? (select all that apply)
Cochrane Library
MEDLINE
Embase
HMIC
CINAHL
TRIP
Psycinfo
Open web (Google Scholar, etc)
Other (please specify)

* 14. For the search task you perform MOST FREQUENTLY, what was the SHORTEST amount of time (hours) it has taken?

* 15. For the search task you perform MOST FREQUENTLY, what is the AVERAGE amount of time (hours) it takes?

* 16. For the search task you perform MOST FREQUENTLY, what was the LONGEST amount of time (hours) it has taken?

* 17. For the search task you perform MOST FREQUENTLY, what was the MINIMUM number of queries (or strategy lines) you have used?

* 18. For the search task you perform MOST FREQUENTLY, what is the AVERAGE number of queries (or strategy lines) you use?

* 19. For the search task you perform MOST FREQUENTLY, what was the MAXIMUM number of queries (or strategy lines) you have used?

* 20. To formulate a complete database search, what was the SHORTEST amount of time (minutes) ithas taken?

* 21. To formulate a complete database search, what is the AVERAGE amount of time (minutes) it takes?

* 22. To formulate a complete database search, what was the LONGEST amount of time (minutes) it has taken?

* 23. For the search task you perform MOST FREQUENTLY, who else is usually involved in the process?

* 24. Do you use previous queries or templates to help formulate your search strategy?
Always Often Sometimes Rarely Never N/A

25. With whom do you typically share your search strategies?
Nobody
Colleagues in my workgroup
Colleagues across my organisation
Public forums
Other (please specify)

Query formulation

* 26. Please indicate the level of your agreement to each of the following statements.
Strongly agree Agree Neutral Disagree Strongly disagree N/A
Boolean logic is important to formulate effective queries (e.g. AND, OR, NOT, etc.)
Proximity, Adjacency, or Distance operators are important to formulate effective queries.
Weighting is important to formulate effective queries (e.g. relevance ranking)
Truncation (Left/Right) is important to formulate effective queries.
Wildcard operator is important to formulate effective queries.
Field operators are important to formulate effective queries (e.g. specific fields having specific values)
Query expansion is important to formulate effective queries (e.g. terms are expanded to include synonyms)
Query translation is important to formulate effective queries (e.g. English to French)
Case sensitivity is important to formulate effective queries.
I need to consider abbreviations and acronyms to formulate effective queries.
I need to consider misspellings to formulate effective queries.
I need to consider synonyms and related terms formulate effective queries.

* 27. Which of the following operators do you use?
Always Often Sometimes Rarely Never N/A

AND
OR
NOT
Phrasal operators (e.g.quotes)
Grouping (e.g.parentheses)
Wild cards (e.g. * or ?)
Proximity (e.g. NEAR or ~)
Field specifiers (e.g.title, abstract, etc.)

28. Are there any operators that don’t currently exist but you wish did?

29. Are there any taxonomies/thesauri you regularly use?

* 30. How do you create/edit your search strategy? (please check all that apply)
Manually, on paper
Using a text editor
Using a form-based query builder
Using a visual query builder
Other (please specify)

Evaluating the results

31. What criteria do you apply when evaluating the results?

* 32. What would you consider to be the ideal number of results returned for a typical search task?

* 33. To complete the search task you perform MOST FREQUENTLY, what was the MINIMUM number of results you have examined?

* 34. To complete the search task you perform MOST FREQUENTLY, what is the AVERAGE number of results you examine?

* 35. To complete the search task you perform MOST FREQUENTLY, what was the MAXIMUM number of results you have examined?

* 36. To assess the relevance of a single search result (e.g. article), what was the MINIMUM amount of time (in minutes) you have spent?

* 37. To assess the relevance of a single search result (e.g. article), what is the AVERAGE amount of time (in minutes) you spend?

* 38. To assess the relevance of a single search result (e.g. article), what was the MAXIMUM amount of time (in minutes) you have spent?

* 39. Please add an example of a typical search strategy:

* 40. Which restriction criteria do you apply to narrow down results?
Always Often Sometimes Rarely Never N/A
Published date
Publication language
Publisher/Source
Author
Availability of source data
Availability of publication
Academic affiliation

41. What other restriction criteria do you apply to narrow down results?

* 42. When search results appear, where do you usually click first?
On the first item
On the item which appears to have the most trustworthy source
On the item which looks most relevant
On the most recent item
Other (please specify)

* 43. How often do you look at the second or third page of results?
Always Often Sometimes Rarely Never N/A

* 44. How do you usually decide when your search task is complete?
Always Often Sometimes Rarely Never N/A
You use all the time allotted for the task
You find a specific result
You can't find any new, relevant results
Other (please specify)

* 45. How often do you face situations where you cannot complete a search task?

46. If you face situations where you cannot complete a search task, please say why.

* 47. Considering the task as a whole, what proportion of your time is spent on:
Most Some A little None N/A
Retrieval (constructing and executing the query)
Analysis (examining results, e.g. for relevance)
Sensemaking (deciding what to do with the output)

* 48. Which of the following activities do you engage in when completing a search task?
Always Often Sometimes Rarely Never N/A
Locating: retrieving specific (possibly known) items or results
Verifiying: confirming that an item or result meets some specific criterion
Monitoring: maintaining awareness of the status of something
Comparing: identifying similarities & differences between items or results
Comprehending: generating independent insight by interpreting patterns within results
Evaluating: using judgement to determine the value of an item or result
Exploring: investigating a set of results or data for the purpose of knowledge discovery
Analyzing: examining a set of results or data to identify patterns & relationships
Synthesizing: creating a novel or composite artefact from separate inputs

About the ideal search engine for your search tasks

* 49. Please indicate the level of your agreement to each of the following statements.
Strongly agree Agree Neutral Disagree Strongly disagree N/A
Recency of retrieved results is important for my work
Search history is important for my work
Combining search queries is important for my work
Combining multiple search results is important for my work
Saving custom lists from search results is important for my work
Storing search results with an expiry date is important for my work
Organising search queries is important for my work
Exporting search queries (histories) is important for my work
Alerting function is important for my work
Using search facets/filters is important for my work

50. How could the process of creating and managing search strategies be improved for you?

51. Can you name any professional groups where you might discuss issues related to search strategy formulation?

52. What other features do you consider are important in an ideal search engine?

More about you

53. Your age

54. Gender

55. What is your nationality?

56. What is your native language?

57. What degrees do you have? (select all that apply)
Diploma
Bachelor
Master
PhD
Professional qualifications
Other (please specify)

58. What are the subjects of your degrees? (select all that apply)
Arts
Humanities
Natural science
Social science
Applied science
Professional/vocational
Other (please specify)

59. Do you have any comments on this survey?

60. If you would like to receive a copy of the final report please provide your email address.
